# Qualitative Feedback From a Text Messaging Intervention for Depression: Benefits, Drawbacks, and Cultural Differences

**DOI:** 10.2196/mhealth.3660

**Published:** 2014-11-05

**Authors:** Adrian Aguilera, Clara Berridge

**Affiliations:** ^1^University of California, BerkeleySchool of Social WelfareBerkeley, CAUnited States; ^2^University of California, San FranciscoSan Francisco General HospitalDepartment of PsychiatrySan Francisco, CAUnited States; ^3^Brown UniversitySchool of Public HealthProvidence, RIUnited States

**Keywords:** mobile health, depression, text messaging, culture, digital health, cognitive behavioral therapy, disparities, mental health, behavior change

## Abstract

**Background:**

Mobile health interventions are often standardized and assumed to work the same for all users; however, we may be missing cultural differences in the experiences of interventions that may impact how and if an intervention is effective.

**Objective:**

The objective of the study was to assess qualitative feedback from participants to determine if there were differences between Spanish speakers and English speakers. Daily text messages were sent to patients as an adjunct to group Cognitive Behavioral Therapy (CBT) for depression.

**Methods:**

Messages inquired about mood and about specific themes (thoughts, activities, social interactions) of a manualized group CBT intervention. There were thirty-nine patients who participated in the text messaging pilot study. The average age of the participants was 53 years (SD 10.4; range of 23-72).

**Results:**

Qualitative feedback from Spanish speakers highlighted feelings of social support, whereas English speakers noted increased introspection and self-awareness of their mood state.

**Conclusions:**

These cultural differences should be explored further, as they may impact the effect of supportive mobile health interventions.

**Trial Registration:**

Trial Registration: Clinicaltrials.gov NCT01083628; http://clinicaltrials.gov/ct2/show/study/NCT01083628 (Archived by WebCite at http://www.webcitation.org/6StpbdHuq).

## Introduction

### Health Interventions Using Mobile and Digital Technologies

Mobile and digital technologies provide opportunities for increasing patient engagement and monitoring. Health interventions delivered through text messaging and other telecommunications have shown promising results in a variety of studies addressing varied health issues. Examples of targets include smoking cessation [1-3]; diabetes [4]; treatment of sexually transmitted infections; supporting community-based HIV/AIDS health workers; and improving primary care attendance [5]. In the area of mental health, texting has been used to assess and treat serious mental illness [6], schizophrenia [7], and depression [8]. Text messaging is increasingly seen as a valuable provider tool because it is widely available, used with relatively low cost and ease, can be applied to a range of health and mental health conditions [9], and can reach underserved populations [10]. Latinos and African Americans are more likely than other racial/ethnic groups to gather health information through their phones [11], which suggests that this medium may be an ideal tool to reach these populations that experience detrimental health disparities [12].

While evidence of efficacy of text messaging-based interventions is growing, mobile health research and intervention development are still in early stages. Now it is important to build knowledge about the user experience from the perspectives of diverse populations to understand if and how interventions impact people. While a focus on clinical outcomes is also crucial, these outcomes cannot be achieved if we do not understand how people interact with digital health interventions. For example, there are limited studies that explore the role of culture and culture-specific messaging in digital health interventions [5]. When developing an intervention, particularly one that is standardized utilizing technology, we tend to assume that it will act similarly for all people who use it since they are receiving the same information and experience; however, the information provided via digital health technologies may be perceived differently based on one’s worldview or cultural values, which may modify the experience. A cultural difference that has been the subject of extensive study has been the comparison of the individualistic framework that is common in the United States in contrast to the collectivist framework that is more typical of other parts of the world. An individualist perspective values autonomy and frames the individual as the agent of change and success, whereas a collectivist perspective is relational in nature and emphasizes maintaining harmonious relationships and fitting into a larger social structure [12]. Culture is by no means the only factor that could influence how individuals perceive an intervention, but in a diverse country like the United States, it merits attention along with other factors such as age, gender, socioeconomic status, and education.

### In-Between Session Messaging

An area of clinical practice that could benefit from the use of messaging technologies is the time between psychotherapy visits in order to increase engagement and ultimately improve outcomes [13,14]. In-between session messaging can increase self-awareness, skill building, as well as perceived support. Aguilera and Muñoz [8] reported initial findings on a text messaging adjunct to cognitive behavioral therapy, and noted that Spanish speakers reported feeling support and cared for, while English speakers mainly reported increased self-awareness.

This paper will present findings from qualitative data gathered from patients about their experiences receiving text messages as part of their cognitive behavioral therapy for depression. We will identify positive and negative feedback as well as highlight how culture may play a role in the perception of the text messaging intervention.

## Methods

### Development of the System

The development and initial testing of this system were reported previously in more detail [8]. Patients in a group cognitive behavioral therapy for depression (based on Health Management of Reality, HMOR, and Building Recovery by Improving Goals, Habits, & Thoughts, BRIGHT, manuals) [15,16] in an urban public sector clinic were asked if they wanted to receive text messages as part of their participation in therapy. The live intervention consists of four four-week modules that focus on thoughts, behaviors, interpersonal issues, and health. Patients were asked if they were interested in using text messaging as part of their group therapy to aid in their completion of their “homework”, which consisted of mood monitoring, as well as monitoring of thoughts, activities, social interactions, and healthy activities. Participants were also given the option to continue receiving text messages after they “graduated” from the therapy, which most patients (17/20, 85%) accepted. For those who did not own a phone, we provided a prepaid mobile phone (~US $10) and provided a prepaid service during the study for a cost of US $25 a month that included unlimited phone calls and text messages. We taught participants who did not know how to use text messaging how to respond to messages. If a patient did not know how to text, a clinician or research assistant sat with that person to help them find where the text messages were stored and orient them to the specific functions of their phone. We would also send a sample message and walk them through the process of responding to the message. If they were not successful on the initial try, we would attempt again until they felt comfortable replying. We would then ask them to find a text message from their messages list, and to try to reply to a message that was sent previously. The next week, we would check in with patients who were having difficulty responding, to try to troubleshoot issues and go through the steps again. Participants who still had difficulties after the third try had a difficult time engaging in the intervention. Some were still able to read the messages, but not respond to them. Some patients took to the texting very quickly, while others, especially older patients, demonstrated more difficulty.

Recruitment occurred in two stages with the first half of participants recruited from 2010 through 2011, and the second round recruited from February to December of 2013. During the first round of recruitment, basic messaging included a daily mood monitoring message (“What is your mood right now on a scale of 1-9?”), sent at random times, and a thematic question sent in the evening (8 p.m.), asking patients to report their quantity of positive thoughts, pleasant activities, social interactions, and healthy activities (eg, “How many positive thoughts have you noticed today?”, “How many positive social interactions did you have today?”, etc*)*. These four areas were the focus of the group therapy for four weeks at a time during the 16 weeks of the treatment.

The second round of participants received the same mood question and also received a thematic question or a “tip” that was different everyday, but was consistent with the theme for that month. Additionally, the second round of participants received reminders to attend their therapy session and also had the option of receiving medication reminders if they chose. The groups enrolled participants on a rolling basis, and patients graduated from the group after completing all four modules and after displaying low stable symptom levels; thus, patients sometimes stayed longer than the standard 16 weeks.

After two and four months of receiving text messages, 20 of the 39 patients answered open-ended questions about their positive and negative experiences receiving text messages. This questionnaire also asked them to rate their agreement on a 1-5 scale (do not agree to strongly agree) to two questions asking whether receiving messages made them feel close to the group, and whether receiving messages made them more likely to attend sessions. Those who completed these surveys were significantly older (mean 55.73 vs 49.27), attended significantly more sessions (mean 20 vs 7), and were available to provide feedback in person after using it over time (see Table 1). As shown in Table 1, other demographic variables, including initial Patient Health Questionnaire - 9 (PHQ-9) scores, were not significantly different. There were 15 participants that replied in Spanish and 5 replied in English. Most participants were women (n=13). Of the 5 English speakers, 1 was Latina, 1 was African American, and 3 were Euro-American. All of the Spanish speakers were Latino/a from either Mexico or countries in Central and South America. All patients had a diagnosis of depression and at least one other chronic physical illness (ie, diabetes, cardiovascular disease, chronic pain, etc). Average PHQ-9 scores at the start of therapy were 10.94, which reflects moderate depression symptoms. There were 17 that had their own phones and 12 of the 20 had used text messaging prior to participating in the study. The website used to send and receive automated messages is now called HealthySMS [17].

**Table 1:** Respondent characteristics.

	Respondents	Nonrespondents
	n=20	n=19
Response rate to SMS, %	47	42
Age	55.73	49.27^a^
Sessions attended	20	7.27^a^
Use SMS^b^ prior, n (%)	13/20 (65)	12/19 (63)
Learned SMS^b^ for study, n (%)	7/20 (35)	6/19 (32)
Continued, n (%)	17/20 (85)	0/19 (0)
PHQ9	10.94	10.24

^a^ *P*<.05

^b^ SMS = short messaging service

### Participants Responses to Open-Ended Questions

Participants provided written responses to questions about their experiences after two to four months of receiving the messages. The qualitative study sample was asked open-ended questions about what they liked about receiving text messaging (“What did you find helpful or positive about the text messages you received?”), what they did not like (“What were the downsides of receiving the messages or what did you find unhelpful?”), what they would suggest to improve the text system (“What other kinds of messages would you find helpful?”, “How else might text messaging improve your health care?”), and reasons they did not respond to the texts, when applicable, (“For the times you did not respond, what were some of the reasons?”). These responses were transferred verbatim into Excel, where the data were analyzed for concepts and possible themes using the open coding method of grounded theory by three separate raters. Concepts were delineated and codes were derived from the data and modified throughout this process of reading and rereading the data [18]. All raters discussed disagreements in ratings until all three reached a consensus. This inductive process enables the data to speak and codes to emerge organically. Through this open coding process, themes were identified and responses were clustered accordingly. As themes emerged, consistencies and inconsistencies were noted and are described below [19].

## Results

### Helpful, Positive Messages

When we asked participants, “What did you find helpful or positive about the text messages you received?”, their responses clustered around three distinct themes: (1) prompts of self-reflection, (2) feeling cared for and supported, and (3) appreciating having reminders to take medication and to attend their scheduled group therapy sessions. Differences surfaced between the themes reported by Spanish and English speakers. As shown in Table 2, the most commonly reported feedback was that text messages prompted self-reflection and awareness (11/20, 55%). Other feedback included reports of feeling cared for (8/20, 40%), liking reminders (7/20, 35%), being cheered up (6/20, 30%), being behaviorally activated (3/20, 15%), and liking the ease of using texts (1/20, 5%). There were significant differences between language users, with Spanish speakers reporting feeling cared for more often than English speakers. There were no significant differences for other categories.

When comparing English and Spanish speakers with regards to demographic characteristics, there were no statistically significant differences, but it is possible that the detection of differences was limited by a lack of power due in particular to a small English speaking sample. In addition to eliciting qualitative feedback, we also asked patients whether they felt that receiving text messages made them feel closer to the group. Most patients agreed that receiving messages made them feel closer to the group with an average rating of 4.5 (out of 5).

**Table 2:** Language comparison.

		Total	English	Spanish
		N=20	n=5	n=15
Female, n (%)		13/20 (65)	2/5 (40)	11/15 (73)
Response rate, %		47	61	42
Age		56	58	55
Sessions attended		20	27	17
Use SMS^b^ prior, n (%)		13/20 (65)	4/5 (80)	9/15 (60)
Learned SMS^b^ for study, n (%)		7/20 (35)	1/5 (20)	6/15 (40)
Continued, n (%)		17/20 (85)	4/5 (80)	13/15 (87)
PHQ9		10.94	6.2	12.92
Sessions attended		20	27	17
**Average rating (1-5)**				
	Did messages make you feel closer to the group?	4.51	4.00	4.67^a^
	Did messages make you more likely to attend?	4.51	4.00	4.67
**What do you like?**				
	Prompts reflection, n (%)	11/20 (55)	4/5 (80)	7/15 (47)
	Feel cared for, n (%)	8/20 (40)	0/5 (0)	8/15 (53.33^a^)
	Reminders, n (%)	7/20 (35)	0/5 (0)	7/15 (47)
	Cheers you up, n (%)	6/20 (30)	0/5 (0)	6/15 (40)
	Activates (behavioral), n (%)	3/20 (15)	0/5 (0)	3/15 (20)
	Easy, n (%)	1/20 (5)	1/5 (20)	0/15 (0)

^a^ *P*<.05

^b^ SMS = short messaging service

### Self-Reflection and Awareness

Nearly all English speakers reported that they valued the prompts for self-reflection, while only one third of the Spanish speakers valued it. As one English speaker put it,

They made me stop and think for a moment about how I was feeling and why I was having those feelings. My life is so crazy I need a reminder to think about how I feel.

Female English speaking participant

Others noted that it gave them the ability to look “in the proverbial mirror” or that “they forced me to ‘check in’ with myself”. A final example of the self-reflection when receiving a text was provided by a patient who said, “They made me stop and think for a moment about how I was feeling and why I was having those feelings”. The English speakers were highly consistent in their responses that receiving texts increased self-awareness and reflection.

Some Spanish speakers also mentioned benefits of self-reflection. For example, one woman stated that, “Me a ayudado a ser y pensar positivo”, *“*They help me be and think more positively”. Another participant also mentioned self-reflection, but also referred to the group saying,

Me recuerda sobre lo que hablamos en el grupo. Recuerdo de la rehabilitación y de los demàs, (They remind me about what we discussed in group. I remember my rehabilitation and about the other group members.)

Female Spanish speaking participant

These responses from Spanish speakers refer to self-reflection, but as a whole were less focused on self-awareness. They highlighted the fact that receiving messages reinforces the themes of the weekly therapy session and reminds them of the group interaction process. Spanish speakers often mentioned that receiving messages helped them feel better and focus on themselves, yet the theme was not as central as it was for the English speaking participants.

### Perceptions of Support

Differences also appeared in the number of people who mentioned that receiving text messages increased feelings of support. No English speakers offered that they felt supported; however, a majority of Spanish speakers stated that receiving the text messages during the week made them feel cared for and supported. For example, one participant stated, “Te hace sentir que alguien se preocupa por ti”. “It makes you feel like someone is concerned about you”. This suggests that the therapeutic relationship is strengthened via the receipt of the messages. This was echoed by a 41-year-old woman who said she likes “saber que alguien le interesa saber como estuvo mi día y me hace sentir un poco mejor si mi día fue mal”, “knowing that someone cares how my day was makes me feel a little bit better, particularly if I had a bad day”. This feeling of being cared for enabled a sense of hope and support,

Ustedes están poniendo esperanza en que uno siga adelante. Están pendientes. (You are providing hope so that one continues moving forward. You are watching out for us.)

Female participant

Here, the text messages were experienced by users as a way to let their therapists know how they are doing, making the technology an extension of the therapeutic relationship.

A Spanish speaking patient summed up the various positive aspects reported about the intervention, stating, “te hace pensar en ti, te hace sentir que alguien se preocupa por ti, te recuerda tus medicinas, te evalùa constantemente, ayuda a estar active, me impulso a usar un cell”, “it makes you think about yourself, it makes you feel that someone is concerned about you, it reminds you to take your medications, it evaluates you constantly, it motivated me to use a cell phone”.

### Demographic Correlates of Qualitative Themes

In order to assess whether demographic characteristics are related to the type of feedback that patients provided, we conducted correlational analyses of age, sex, language, and previous use of text messaging with the various positive themes that arose. As seen in Table 3, speaking Spanish is related to increased feelings of being cared for, and previous use of text messaging is negatively related to feeling behaviorally activated by receiving text messages.

**Table 3:** Correlations between patient characteristics and qualitative feedback.

	Reflect	Care	Remind	Activates	Easy	Cheer up
Age	0.12	-0.15	-0.06	0.25	0.19	-0.22
Female	-0.03	0.17	-0.34	0.02	-0.31	0.25
Spanish	-0.29	0.47^a^	0.42	0.24	-0.40	0.38
Used SMS^b^ before	-0.33	0.04	-0.04	-.051^a^	0.19	-0.13

^a^ *P*<.05

^b^ SMS = short messaging service

### Downsides of Texting

When the participants were asked about the downsides of receiving text messages, the most common response was that there were none, and that they actually appreciated the messages. An aspect that some participants disliked or had difficulty with was an inability to respond due to being busy (eg, while at work). A patient mentioned, “que interrumpen si estas trabajando”, “they interrupt if you are working”, and another stated that, “sometimes you had a delayed response from me, I might have been preoccupied”.

Another set of problems that arose was technical in nature. These were mostly related to people not being able to access or respond to text messages, “El problema es que no se usar con facilidad los textos, como contestar con mas informacion”. “The problem is that I don’t know how to use text with ease, how to respond with more information”. A way that we attempted to address this problem was that we asked people to only respond with a number to the mood question, while others could also add additional text to provide context to the mood rating. A 70-year-old woman reported, “tenia que leérmelos mi hijo”, “my son had to read them for me”, referring to the difficulty accessing the messages in the phone and to the small text.

When we asked participants, “What other kinds of messages would you find helpful?”, “How else might text messaging improve your health care?”, the vast majority reported “none” or “nothing else”. Others had suggestions for specific messages such as suggesting free or inexpensive activities in the community, as well as varied messages that were less repetitive. A patient even made a list of motivational messages that he suggested including in the set of messages (eg, “Vive tu vida, no vivas la vida de los demás”. “Live your life, don’t live the life of others”. “Confía en ti mismo, ese es el primer paso de tu éxito”. “Believe in yourself, it is the first step in your success”.).

## Discussion

### Qualitative Feedback From Participants

The qualitative feedback obtained from participants in a text messaging intervention for depression showed that the intervention yielded positive experiences that resulted in reports of self-awareness, skill building, and feelings of being cared for and supported. The main criticisms of texting included receiving messages at inopportune times and some technical difficulties for those that were unfamiliar with the technology.

### Cultural Differences

An interesting cultural difference arose when comparing the responses about what was deemed positive about receiving text messages. Spanish speakers more often mentioned that receiving messages made them feel cared for and supported, while English speakers never mentioned this benefit, but stated that the messages were a way to help them be more aware of their mood states. A possible explanation for this difference is that Spanish speakers may place a stronger value on collectivism and relational connections than English speakers, who may value agency and independence more [20]. Another possibility is higher educational levels among English speakers may result in more exposure to technology and different understandings of how technology works and who the messages are coming from, even though all participants were told that messages were sent automatically and not by individuals. Higher levels of education confer increased health literacy [21], as well as familiarity with technology [5]**.** Unfortunately, we do not have systematic data assessing educational background; however, we can note that at least two of the English speakers had a college education and all had at least a high school level of education compared to Spanish speakers, who mostly had only an elementary level education (most only to 6th grade).

The role of supportive accountability in technology interventions has previously been discussed with technological interventions [22]; however, the role of culture in moderating the support felt by users of technological interventions has not been considered. This is an interesting difference that should be explored further, as this is a limited sample, but points to the potential of culture as a moderating influence. Technology-based interventions should assess cultural and demographic differences that may shape the user’s experience. This knowledge can also help further personalize interventions and possibly increase engagement. It is also possible that the intervention may have felt equally supportive cross-culturally by the participants, but that support is communicated more among people who are more collectivistic. Even that difference may show the difference in salience of support cross culturally.

Problems that users experienced included not being able to respond to a message because they were busy, as well as technical difficulties. These are important limitations to consider, but they are also addressable. The issue of bad timing of messages can potentially be solved by narrowing the window at which messages are sent with a feature that enables clients to block off times when text messaging cannot come through, so that they are not bothered when they know they do not want to be interrupted. The issue of technical difficulties, such as the inability to access messages and making messages basic and readable, are more challenging. Some ways to address these problems included keeping the messages and response options simple, so that individuals at various skill levels can still participate. The increased ubiquity of smartphones will also aid in this, as there are more accessibility friendly features that can enlarge or even read text out loud. A continuing challenge is making technology-based interventions available to those with limited tech-savviness and literacy.

### Limitations

The data were collected from participants who stayed in therapy long enough to provide responses in person, so results are skewed toward those who are engaging in therapy. People who attended one session and did not return did not provide feedback, which could be helpful for highlighting more of the negative aspects of receiving text messages.

The sample size was small, which limits generalizability. In particular, the number of English speakers was only 5, compared to 15 Spanish speakers, which reduces the confidence in the language differences. However, the consistency of English speaking respondents at least merits further study of this question in future research.

Another potential limitation is the focus on text messaging, which is a basic technology. The lessons we are learning from the use of standard text messaging can transfer to other messaging platforms and applications for use when smartphones are more ubiquitous. For instance, the crowdsourcing of messages could be an effective precedent in delivering personalized messages going forward. The fact that most people in this study did not provide suggestions to improve the system may reflect a lack of knowledge of the potential of technology. Many people may not realize the possibilities of technology and are limited in providing suggestions, but those who do provide suggestions are a potentially valuable resource.

### Conclusions

Based on our developmental research, we have found that it is useful to take the time to teach people how to use the technology and see them be successful. It is also important to keep things simple so that more people can engage. Text messaging is simple enough for people to engage in for long periods of time, and it can provide important information for clinicians. Interventions can also have tiered levels of interaction. For example, we asked for mood, but gave the option to add additional context about that mood rating. Some people provided this, while others did not. Responding with a number is relatively simple, but adding text is more complicated, particularly with older phones or for people with eyesight difficulties. People with less previous use of technology have a higher barrier to entry, but may be more likely to engage and get more out of technological tools. Learning to use a new technology seems to build self-efficacy, and it is still a novel task with a high likelihood of engagement as long as they pass the threshold of feeling comfortable with using it.

It is important to ask about what people like and dislike about a digital health intervention. This helps steer developers of the technology to possible mechanisms of action in an intervention. Technology-based adjuncts seem to be extensions of a clinical and therapeutic relationship. Just as there are cultural nuances in the delivery of live psychosocial interventions [23], technology-based interventions should also consider the role of culture in shaping perceptions. It is likely that the relationship that a clinician has with their clients will serve as the basis for engagement with technology. Text messages may also be a particularly salient medium of communication for depressed Latinos, as they report feeling isolated and receiving a message reminds them that someone cares.

The results from this study show that there is potential for utilizing digital health technologies such as text messaging as an adjunct to mental health practice, but that people may experience digital health interventions differently based on demographic and cultural differences. It is important to take these diverse values into account as we disseminate digital health interventions to underserved and low-income populations that have high need.

## Abbreviations

PHQ-9: Patient Health Questionnaire - 9
